# Supplementary material for: Complement C3d enables cell-mediated immunity capable of distinguishing spontaneously transformed from nontransformed cells
Source: Proc Natl Acad Sci U S A. 2024 Dec 18;121(52):e2405824121. doi: 10.1073/pnas.2405824121 (PMC11670236; doi:10.1073/pnas.2405824121)
Supplement: Supplementary file 14 — Dataset S13 (RTF) [file pnas.2405824121.sd13.rtf]

Assay ID	Availability	Catalog Number	Assay Type	Gene Symbol	Gene Name	Alias	RefSeq	GenBank mRNA	Species	Amplicon Length	Best Coverage	3' Most	Matched Keywords		
Mm00432939_m1	Inventoried	4453320	GE	E2f1	E2F transcription factor 1	E2F-1;mKIAA4009	NM_007891.5;NM_001291105.1	L21973.1;AF483514.1;AK153968.1;BC052160.1;AK220226.1;AF483515.1;AK017841.1	Mouse	81	Yes	No	All		
Mm00485586_m1	Inventoried	4453320	GE	Rb1	retinoblastoma 1	Rb;Rb-1;pRb	NM_009029.2	BC096525.1;AK132875.1;AK141028.1;AK146467.1;M26391.1;DQ400415.1	Mouse	80	Yes	No	All		
Mm00438063_m1	Inventoried	4453320	GE	Ccna2	cyclin A2	AA408589;Ccn-1;Ccn1;Ccna;CycA2;Cyca	NM_009828.2	BB642910.1;BC052730.1;Z26580.1;AK216847.1;X75483.1;AK188335.1;AK135861.1;AK044924.1	Mouse	83	Yes	No	All		
Mm03009468_g1	Inventoried	4448892	GE	Tfdp1	transcription factor Dp 1	Dp1;Drtf1	NM_001291766.1;NM_001291768.1;NM_001291765.1;NM_009361.3	AK030820.1;BC145344.1;AK013180.1;AK154037.1;BC132569.1;BC132567.1;BC058633.1;X72310.1	Mouse	67	No	No	All		
Mm00432337_m1	Inventoried	4448892	GE	Ccna1	cyclin A1		NM_007628.3	BC125436.1;X84311.1;BC120518.1;AK077114.1	Mouse	59	Yes	No	All		
Mm00618407_m1	Inventoried	4448892	GE	Tfdp2	transcription factor Dp 2	1110029I05Rik;A330080J22Rik;DP-3;DP3	NM_001184711.1;NM_001184709.1;NM_001184710.1;NM_178667.4;NM_001184706.1;NM_001184708.1	AK044784.1;AK029055.1;AK158829.1;BC141080.1;AK033356.1;BC092230.1;CA980067.1;AK053881.1;AK079624.1	Mouse	78	Yes	No	All		
Mm02745760_g1	Inventoried	4448892	GE	Hdac1	histone deacetylase 1	HD1;Hdac1-ps;MommeD5;RPD3	NM_008228.2	U80780.1;AK194630.1;BC092070.1;X98207.1;BC108371.1;AK076080.1	Mouse	74	Yes	Yes	All		
Mm00443947_m1	Inventoried	4453320	GE	Cdk2	cyclin-dependent kinase 2	A630093N05Rik	NM_183417.3;NM_016756.4	AK154997.1;AK153983.1;BC026145.1;U63337.1;AK146845.1;AK148017.1;AK152922.1;BC005654.1;AK042462.1;AK200296.1;AK151979.1;AJ223732.1	Mouse	62	Yes	No	All		
Mm01239040_s1	Made to Order	4448892	GE	Mycs	myc-like oncogene; s-myc protein		NM_010850.2	BC120777.1;AK132789.1;BC117074.1	Mouse	67	Yes	No	All		
Mm01242468_m1	Inventoried	4448892	GE	Rbl2	retinoblastoma-like 2	PRB2;RBR-2;Rb2;p130	NM_001282001.1;NM_001282000.1;NM_011250.4	AK160027.1;U50850.1;BC020528.1	Mouse	68	Yes	No	All		
Mm01164793_m1	Made to Order	4448892	GE	4930426D05Rik	RIKEN cDNA 4930426D05 gene		NM_001271580.1	AK015205.1	Mouse	57	No	No	All		
Mm02745694_m1	Made to Order	4448892	GE	4933427I04Rik	Riken cDNA 4933427I04 gene			AK133427.1	Mouse	65	Yes	Yes	All		
Mm01218635_m1	Made to Order	4448892	GE	9230114K14Rik	RIKEN cDNA 9230114K14 gene										
